# Mechanical energy flow analysis in athletes with and without anterior cruciate ligament reconstruction during single-leg drop landing

**DOI:** 10.1038/s41598-024-51631-5

**Published:** 2024-01-15

**Authors:** Hamidreza Zanguie, Rahman Sheikhhoseini, Mohammad Yousefi, Julie A. Hides

**Affiliations:** 1 Department of Corrective Exercise and Sport Injury, Faculty of Physical Education and Sport Sciences, Allameh Tabataba’i University, Tehran, Iran; 2 Department of Sports Biomechanic, Faculty of Physical Education and Sport Sciences, University of Birjand, Birjand, Iran; 3 School of Health Sciences and Social Work, Griffith University, Gold Coast, QLD Australia; 4 Menzies Health Institute Queensland, Griffith University, Gold Coast, QLD Australia

**Keywords:** Physiology, Anatomy

## Abstract

Techniques that reduce mechanical energy have been linked to lower chances of experiencing an Anterior Cruciate Ligament (ACL) injury. Although there is evidence that movement patterns are altered in athletes who have undergone Anterior Cruciate Ligament Reconstruction (ACLR), energy transfer mechanisms have not been examined. This study aimed to compare energy flow mechanisms during single-leg drop landing between athletes with and without history of ACLR. A total of 20 female athletes were included in this study. Ten participants underwent ACLR 12 months ago (mean age, 21.57 ± 0.41 years) and 10 were healthy controls (mean age, 20.89 ± 0.21 years). Participants executed the single-leg drop landing (SLL) maneuver by descending from a 30 cm wooden box and landing on the tested leg on an embedded force plate. Information collected during the SLL trials was refined using rigid-body analysis and inverse dynamics within Nexus software, ultimately allowing construction of skeletal models of the athletes. Ankle and knee mechanical energy expenditure (MEE) was higher in the control participants during landing. However, the result for the hip MEE demonstrated that MEE of the control group was significantly lower compared with the ACLR group, but MEE of the control subjects was higher as compared to ACLR group (p ˂ 0.05). Results suggest the avoidant use of the quadriceps muscle post ACLR leads to knee-avoidant mechanics and loss of knee joint power generation during a SLL task.

## Introduction

The rate of primary Anterior Cruciate Ligament (ACL) rupture in athletes is 1 in 3500^1^. The prevalence of recurrent ACL rupture is high, and ranges from 1 to 11%^2^. Additionally, the prevalence of secondary contralateral ACL injury is as high as 1 in 4^3^, which suggests an elevated risk of ACL injury among athletes who have undergone a, ACLR^4–6^.

There are several factors thought to contribute to secondary ACL injuries^7,8^. The most significant biomechanical factors are kinematic, neuromuscular and kinetic. Biomechanical factors include reduced knee flexion range of motion, increased internal rotation angle and valgus of the knee and decreased hip flexion and increased hip adduction^9,10^. Neuromuscular factors include smaller increases in activation of the vastus medialis, vastus lateralis, gastrocnemius lateralis, and changes in hamstring activation and co-contraction^6,11^. Kinetic factors include changes in the ground response forces and a decrease in the extensor torque of the knee and ankle^12,13^ during dynamic activities including squat, single-leg drop landing (SLL), gait, and running^5^. To control joint motion and absorb the body's kinetic energy during landing, internal rotation of the hip, knee, and plantarflexion of the ankle moments must be created by eccentric muscle contractions^14^.

It appears that movement changes present in healthy participants or subjects with injuries may predispose them to further injuries based on the kinesio-pathologic model of musculoskeletal pain disorders^15,16^. Following a ACLR, a number of biomechanical changes influence joint motion of the lower limbs^17^. It is said that any changes made by the system as a result of pain or pathology mirror any changes in the sensorimotor system and result in systemic and predictable patterns^18^. There may be hidden causes for many indications and symptoms of musculoskeletal system dysfunction that are present elsewhere^19^. Hence, the idea of chain reactions stresses the clinical notion of seeing past the location of the pain and concentrating on its cause rather than its source^20^. In order to provide proper evaluation and therapy, physicians need to be able to comprehend and predict the progression of functional impairments. Despite the detection of these biomechanical adaptations in previous studies, the high rate of secondary ligament injury is persists^21^. Therefore, the main possible causes of ACL injury recurrence remain unclear.

Compensatory movement patterns of the lower limb joints have been observed during functional tasks and activities^13,22^. These compensatory mechanisms may contribute to the asymmetry observed in limbs post ACLR and increase the stress load on the lower limb joints. They may also predict the risk of secondary ACL injury^23^. These results suggest that the probability of subsequent ligament injury increases with increasing vertical ground reaction force. Hence, studying single-leg drop landing biomechanics could provide useful information^24^. Subsequent ACL injury may be able to be predicted by the state of the hip, knee, and ankle joints. To prevent secondary ACL injury, altered biomechanical adaptations should be studied^4,6^ as a possible crucial biomechanical chains, but, these adaptations commonly have been examined locally in every joint. Thus, the ability to control joint motion and absorb the body's kinetic energy during landing is particularly important for athletic populations given the high physical demands of sport.

Energy can be transferred between adjoining segments during dynamic tasks to absorb energy and minimize the loading rate to decrease possible tissue damage. Although there is evidence that movement patterns are altered in athletes who have undergone ACLR, energy transfer mechanisms have not been examined. It seems that energy transfer mechanisms are change in musculoskeletal disorders^25^. Moreover, there is lack of evidence regard to energy transfer mechanisms in elite female athletes with history of ACLR. Therefore, the current study aimed to compare energy flow mechanisms during single-leg drop landing between athletes with and without a history of ACLR.

## Methods

### Study design

The current study was a cross-sectional study.

### Participants and setting

Twenty elite female athletes who previously competed for Iran in taekwondo league first divisions or national teams participated in this cross-sectional study. Based on previous research^26^ and utilizing G*Power version 3.1 software, with a significance level (α) of 0.05 and a power (β) of 0.10, using independent samples *t*-tests, a total sample size of 20 was required for this study. Based on the convenience sampling method, we included a total of 10 females with ACLR and 10 females without a history of ACL injury. Participants were referred to the biomechanics laboratory of Shahid Beheshti University of medical sciences, Tehran. Ethics approval for this research was obtained from the Research Ethics Committee of Allameh Tabataba’i University, Tehran (Ethical code: IR.ATU.REC.1400.030).

For the ACLR group, the surgery was carried out using a medial hamstring autograft performed at least 12 months prior to the study in their dominant leg (preferred leg to kicking the ball). All participants were right leg dominant. Athletes who had undergone a revision of their ACLR surgery, had concurrent knee ligament surgery, repairable meniscal damage, full-thickness articular cartilage damage or a history of a major lower extremity injury (such as a fracture were excluded)^26^. The participants were then given oral and written information about the study's objectives and procedures at a familiarization session and all athletes provided informed consent before participating in the study. For non ACLR group, female athletes who had no previous history of knee meniscal or ACL injuries were recruited. They were excluded if they had the history of lower extremity injuries during the last 3 months that resulted in at least one-day loss of training or competition, any history of orthopedic surgery in the lumbopelvic or lower extremities, or any current medical conditions that they need for medications.

### Data sources

The analysis of the three-dimensional (3D) motion in the SLL task was conducted utilizing a configuration of eight digital cameras (VICON: 8 Vero; 2 Vantage, Oxford, UK). The kinematics of the joints were recorded at a frequency of 100 Hz, employing 36 retro-reflective markers placed strategically on specific anatomical landmarks such as the anterior superior iliac spine (ASIS) and posterior superior iliac spine (PSIS), femoral condyles and malleoli, heels, and the base of the first, second, and fifth metatarsals. These markers facilitated the determination of joint centers and segments of the lower extremities^27,28^. Additionally, four rigid-shell clusters were affixed to the athletes' thighs and legs to monitor the motion of these segments. Concurrently, kinetic data was collected using a force plate embedded in the ground. This plate captured ground reaction forces (FP4060-08, Bertec Corp., Columbus, OH, USA) at a sampling rate of 1000 Hz.

The athletes executed three attempts of the SLL task from a height of 30 cm, using the ACLR limb. Prior to the actual testing, the athletes were provided instructions on how to perform the SLL task, and each athlete underwent several practice trials^29^. In the SLL task, athletes descended from a 30 cm wooden box, landing the tested limb onto a ground-mounted force plate^29^ while they were asked to maintain their hands on the hips. A trial was deemed successful if the athlete left the wooden box with both feet simultaneously, managed to land on the force plate with the tested limb, and maintained their balance^30^. The information obtained from the SLL trials underwent further processing through rigid-body analysis and inverse dynamics within Nexus software (v2.7, Vicon, Oxford, UK) to construct skeletal models representing the athletes. The data of dominant legs were analyzed for all participants. A fourth-order low-pass Butterworth filter with a cutoff frequency of 10 Hz was applied for filtering data. Joint moments were normalized by dividing them by the product of the athlete's mass and height (Nm/kg m).

Net joint forces (F-sources) and torques (T-sources) which are in charge of the motion that is being seen are the genuine sources of mechanical power that must be analyzed in order to transmit mechanical energy between body segments. The joint should have equal torques and reaction forces acting in opposite directions, according to Newton's third law (${T}_{j}^{p}={-T}_{j}^{d}{\mathrm{ and }}{F}_{j}^{p}={-F}_{j}^{d}).$ J represents the joint, d means the distal segment and p represents the proximal segment. All proximal joint torques were considered as positive values, and distal joint torques were considered as negative values. Net joint power (P_j_) from which mechanical energy expenditure MEE (U_j_) is calculated^25^:

1 $${P}_{j}= {T}_{j}^{p}\left({\omega }^{p}-{\omega }^{d}\right) = T_{j}^{p}{\omega }^{p}-{T}_{j}^{d}{\omega }^{d}={P}_{p}+{P}_{d}$$

2 $${U}_{j}={\int }_{{t}_{1}}^{{t}_{2}}\left|{P}_{j}\right|dt$$

Aleshinsky introduces the concept of transferring mechanical energy (referred to as flow energy) within the context of single joint muscle actions^31–34^. Table 1 outlines all conceivable power flow situations or modes. Modes 1 and 4 (Table 1) signify scenarios in which adjoining links rotate in opposing directions, and muscles are not counterbalanced by segmental energy. Conversely, modes 2, 3, 5, and 6 (Table 1) symbolize situations where both segments rotate in the same direction, thus offsetting the effects of muscles^33^. The calculation of mechanical energy compensation (MEC) involves dividing the overall joint mechanical energy expenditure (G_j_) by the net joint mechanical energy expenditure.

**Table 1:** Kinematic and kinetic conditions associated to flow energy modes.

Power flow mode and conditions		Torque/velocity conditions	Description
1	${P}_{p}<0$, ${P}_{d}<0$, ${P}_{j}<0$	${T}_{j}^{p}>0$, ${\omega }^{p}<0$, ${\omega }^{d}>0$	The muscles absorb all energy, with the distal and proximal segments losing power
		${T}_{j}^{p}<0$, ${\omega }^{p}>0$, ${\omega }^{d}<0$	
2	${P}_{p}<0$, ${P}_{d}>0$,${P}_{j}<0$	${T}_{j}^{p}>0$, ${\omega }^{p}<0$, ${\omega }^{d}<0$, and $\left|{\omega }^{p}\right|> \left|{\omega }^{d}\right|$	The muscle absorbs the surplus power that exceeds what reaches the distal segment from the proximal segment
		${T}_{j}^{p}<0$, ${\omega }^{p}>0$, ${\omega }^{d}>0$, ${\mathrm{and }}\left|{\omega }^{p}\right|> \left|{\omega }^{d}\right|$	
3	${P}_{p}<0$, ${P}_{d}>0$,${P}_{j}>0$	${T}_{j}^{p}>0$, ${\omega }^{p}<0$, ${\omega }^{d}<0$, and $\left|{\omega }^{p}\right|<\left|{\omega }^{d}\right|$	The muscles are responsible for the extra power that enters the distal segment and exits the proximal segment
		${T}_{j}^{p}<0$, ${\omega }^{p}>0$, ${\omega }^{d}>0$, and $\left|{\omega }^{p}\right|< \left|{\omega }^{d}\right|$	
4	${P}_{p}>0$, ${P}_{d}>0$,${P}_{j}>0$	${T}_{j}^{p}>0$, ${\omega }^{p}>0$, ${\omega }^{d}<0$	Power enters the proximal and distal segments; the muscles are the only source of energy
		${T}_{j}^{p}<0$, ${\omega }^{p}<0$, ${\omega }^{d}>0$	
5	${P}_{p}>0$, ${P}_{d}<0$,${ P}_{j}>0$	${T}_{j}^{p}>0$, ${\omega }^{p}>0$, ${\omega }^{d}>0$, and $\left|{\omega }^{p}\right|> \left|{\omega }^{d}\right|$	The proximal segment receives more power than the distal segment does, and the extra power is generated by the muscles
		${T}_{j}^{p}<0$, ${\omega }^{p}<0$, ${\omega }^{d}<0$, and $\left|{\omega }^{p}\right|> \left|{\omega }^{d}\right|$	
6	${P}_{p}>0$, ${P}_{d}<0$,${ P}_{j}<0$	${T}_{j}^{p}>0$, ${\omega }^{p}>0$, ${\omega }^{d}>0$, and $\left|{\omega }^{p}\right|< \left|{\omega }^{d}\right|$	The muscles absorb the surplus power that escapes from the distal segment and enters the proximal segment
		${T}_{j}^{p}<0$, ${\omega }^{p}<0$, ${\omega }^{d}<0$, and $\left|{\omega }^{p}\right|< \left|{\omega }^{d}\right|$	

3 $${G}_{j} = 1-\frac{{\int }_{{t}_{1}}^{{t}_{2}}\left|{P}_{j}\right|dt}{{\int }_{{t}_{1}}^{{t}_{2}}\left[\left|{P}_{d}\right|+\left|{P}_{p}\right|\right]dt}$$

Joint MEE variables were broken down into three transfer conditions: concentric energy transfer (${MEE}_{(+)}={MEE}_{\left(mode3\right)}+{MEE}_{(mode5)}$), eccentric energy transfer (${MEE}_{(-)}=\left|{MEE}_{\left(mode2\right)}+{MEE}_{\left(mode6\right)}\right|$) and no energy transfer (${MEE}_{0}=\left|{MEE}_{\left(mode1\right)}\right|+{MEE}_{\left(mode4\right)}$). Moreover, the MEC was determined independently for eccentric and concentric energy transfer. It should be remembered that the MEC for non-transferable times is always zero.

### Statistical methods

We conducted inferential analysis and descriptive statistics using SPSS version 21. The Shapiro–Wilk test was used to check the data's normality distribution. We used an independent *t*-test to assess the mean differences of the research variables across groups with and without ACLR for all variables, including demographics and eccentric and concentric mechanical energy compensation, as all data were normally dispersed. Effect sizes were reported based on the Partial Eta Squared values. A 95% (0.05) level of confidence was used for all analyses.

### Ethics approval and consent to participate

Prior to starting the investigation, study approval was obtained from the Biomedical Research Ethics Committee of Allameh Tabataba’i University (Ethics code: IR.ATU.REC.1400.030), and all participants gave written informed consent. Authors confirm that all research was performed in accordance with relevant guidelines/regulations.

## Results

Twenty athletes participated in this study. Table 2 provides an overview of the participant's demographic information. No differences in group demographics were observed.

**Table 2:** Demographic characteristics of athletes in ACLR (N = 10) and control (N = 10) groups.

Characteristic	Group	Mean ± standard deviation	p-value
Age, y	Control	20.89 ± 0.21	0.164
	ACLR	21.57 ± 0.41	
Height, cm	Control	164.81 ± 0.69	0.406
	ACLR	165.48 ± 0.37	
Mass, kg	Control	62.8 ± 0.92	0.314
	ACLR	61.56 ± 0.76	
Body Mass Index, kg/m^2^	Control	23.13 ± 0.4	0.186
	ACLR	22.47 ± 0.25	

*ACLR* anterior cruciate ligament reconstruction.

**Figure 1:**
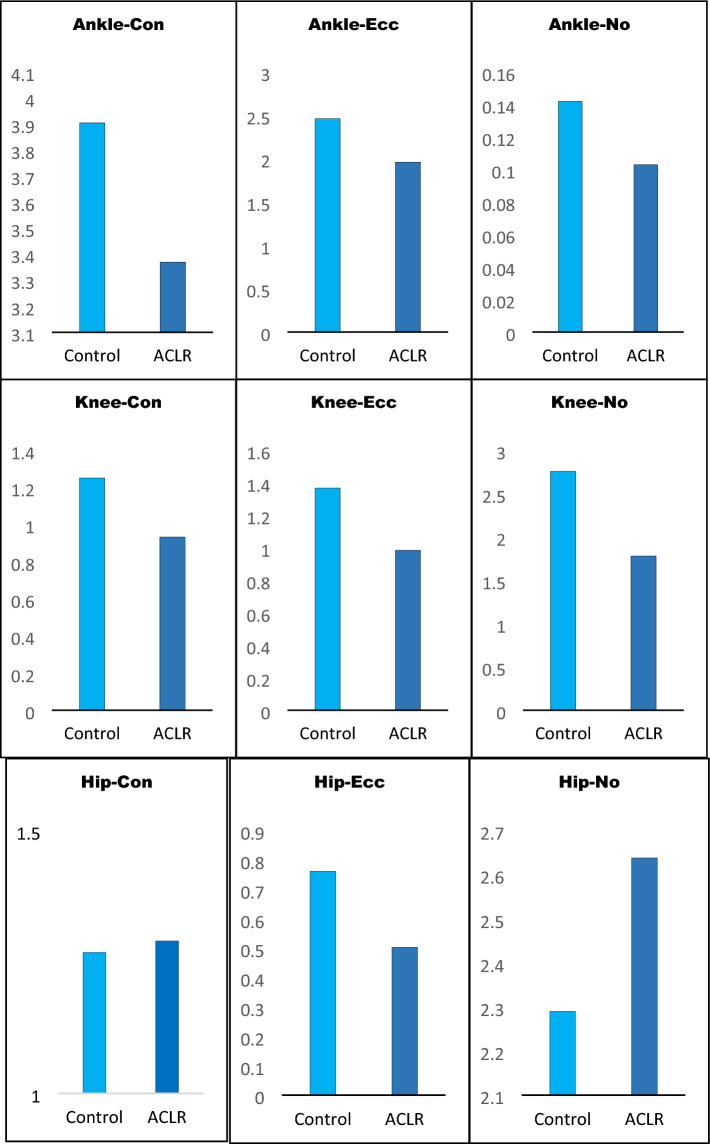
Mechanical energy expenditure during SLL. Standard deviation from the mean is represented by the error bars. *Con* concentric, *Ecc* eccentric, *No* no transfer, *ACLR* anterior cruciate ligament reconstruction.

Independent *t*-tests were used to assess the eccentric and concentric mechanical energy compensation coefficients between female participants with and without ACLR. Ankle and knee MEE were higher in control participants. For the ankle and knee, data analyses indicated that the control group had higher MEE^Ankle and knee^_(+) (−) and (0)_ compared with the ACLR group during landing. The result for the hip MEE demonstrated that the MEE^hip^_(+) and (0)_ of the control group was significantly lower than those with a history of ACLR, but MEE^hip^_(−)_ of the control subjects was higher for the ACLR group (Table 3, Fig. 1).

**Table 3:** Means, standard deviations (SD) and p-value of Mechanical Energy Expenditure (MEE) are presented for control and ACLR groups.

Energy transfer condition (100*J/kg)	Control group	ACLR group	t	p-value	Effect size	95% Confidence interval of the difference	
	Mean ± SD	Mean ± SD				Upper	Lower
Ankle MEE							
Concentric	3.91 ± 0.17	3.37 ± 0.06	9.36	˂ 0.001*	0.830	0.42	0.66
Eccentric	2.46 ± 0.13	1.96 ± 0.14	7.28	˂ 0.001*	0.746	0.36	0.64
No transfer	0.14 ± 0.04	0.10 ± 0.03	2.34	0.03*	0.233	0.00	0.07
Knee MEE							
Concentric	1.25 ± 0.12	0.93 ± 0.09	6.78	˂ 0.001*	0.718	0.22	0.41
Eccentric	1.37 ± 0.26	0.99 ± 0.07	4.41	˂ 0.001*	0.523	0.20	0.56
No transfer	2.76 ± 0.33	1.78 ± 0.43	5.70	˂ 0.001*	0.643	0.62	1.34
Hip MEE							
Concentric	1.27 ± 0.13	1.29 ± 0.07	− 0.52	0.608	0.015	− 0.12	0.07
Eccentric	0.76 ± 0.11	0.50 ± 0.09	5.57	˂ 0.001*	0.633	0.16	0.36
No transfer	2.29 ± 0.19	2.64 ± 0.13	− 4.76	˂ 0.001*	0.557	− 0.50	− 0.19

*ACLR* anterior cruciate ligament reconstruction, *MEE* mechanical energy expenditure, *SD* standard deviation. *Statistically significant differences were observed.

## Discussion

The aim of this study was to compare energy flow mechanisms during landing between athletes with and without ACLR. Our results demonstrated that the ankle and knee MEE were higher in control participants than those who had undergone ACLR. The control participants had higher MEE^Ankle and knee^_(+) (−) and (0)_, lower hip MEE^hip^_(+) and (0),_ and higher hip MEE^hip^_(−)_ when compared with the ACLR group.

As demonstrated in a previous study^35^ in male participants, our research revealed noteworthy decreases in knee joint power within the ACLR group. Regarding the distribution of joint power, we discovered that the dominant limbs that underwent ACL reconstruction exhibited a significantly lower proportion of power coming from the knee joint and a higher proportion originating from the hip joint, in comparison with both their unaffected counterparts and the dominant limbs of healthy individuals. Interestingly, the mean peak concentric hip joint power of ACLR limbs was not different from that of healthy controls (Fig. 1). These findings suggest that the elevated hip joint contribution observed in ACLR limbs might not stem from a shift of effort towards the hip, as implied by prior studies^36^. Instead, the hip joint of the ACLR limb seemed to contribute a greater relative proportion the overall limb power due to substantial power losses noted at the knee and ankle joints. The notable declines in peak knee joint power observed in ACLR limbs were linked to deficiencies in quadriceps strength^37^. Earlier research has demonstrated that the uninvolved limb undergoes changes in jumping kinematics following ACLR; however, the driving factors behind these biomechanical alterations in performance remain unknown^38^.

The discoveries concerning changes in joint power during the SLL task provide intriguing insights into how landing performance is affected following ACLR. However, the availability of motion capture and advanced biomechanical movement analysis is limited for the majority of clinicians and patients. Defining specific cutoff values for quadriceps strength that can predict knee biomechanics during dynamic athletic tasks could offer valuable guidance for making more objective decisions about advancing through higher-level rehabilitation stages. Currently, there is no consensus regarding the appropriate timing for patients to engage in demanding sports activities like landing and sprinting, and most clinicians primarily rely on limb symmetry index measurements to gauge readiness^39,40^. Moreover, it seems that clinicians should train athletes with ACLR history on dynamic landing strategies, training proprioception, neuromuscular control, and mechanics with functional activities.

The landing of athletes with ACLR is significantly affected by the decreased energy expenditure and restricted capacity to make up for the effort performed by the muscles through energy transfer. Subjects with ACLR had a lower overall MEE at the ankle and knee than control participants, which may have been due to an effort to increase stability during the landing task. Athletes with ACLR generate more energy at the hip joint during landing than the control group, and this energy generation (without transfer) may cause kinematic changes during landing^41^. This trend could indicate a transition in the transfer of energy from the knee to the hip joint within the ACLR group, a phenomenon absents in the control group. The outcomes of this investigation propose that assessing the role of joints in the overall mechanical energy expenditure during single-leg drop landing could serve as a valuable method for identifying patients who might undergo compensatory biomechanical adjustments following ACL reconstruction.

The disability framework formulated by Nagi^42^ and elaborated on by additional researchers^43,44^, underscores the significance of pinpointing limitations in mobility as a preventive measure against disability and subsequent injury. As mobility limitations often lead to the adoption of compensatory movement approaches, the recognition and comprehension of these strategies could play a pivotal role in formulating efficient intervention strategies. Investigating the interactions between body segments and muscles while moving allows for the use of mechanical energy^31–34^. We investigated the application of mechanical energy techniques to detect compensatory approaches employed by individuals with ACLR during SLL. While joint power indeed captures the overall impact of a muscle group on the mechanical energy of the whole body, it falls short in fully elucidating the muscle group's contribution to altering the energy status of specific body segments. The localized ramifications of energy transfer can far surpass the net joint power in magnitude and may even exhibit contrasting signs. The data indicated that negative joint power could actually raise segmental energy, while positive joint power might lead to a reduction in segmental energy. Overall, the current study showed that athletes with ACLR showed less energy transfer, absorption and production during SLL than control participants.

At different rates and years after ACLR, quadriceps strength deficits have been documented^45–47^. Despite the fact that the highest quadriceps strength losses are often noted in the first 6–12 months after surgery^48,49^, deficits of between 5 and 18% of the unaffected limb have been noted between 5 and 15 years after ACL restoration and intensive rehabilitation^50^. When compared with healthy matched controls, these quadriceps strength reductions following ACLR are reportedly somewhat bilateral. It has been proposed that reduced shock absorption during SLL and quadriceps avoidance gait patterns may be risk factors for chronic joint pathologies after ACL injury^51^. According to Nyland and colleagues, an ACL reconstructed knee may have altered neuromuscular control methods because there was an 11% decrease in quadriceps strength and a 7% increase in hamstring strength^52^.

Neuromuscular control is commonly defined as involuntary muscle engagement that contributes to maintaining dynamic stability within the joints^53^. After an ACL injury, issues related to quadriceps neuromuscular function, such as muscle weakness, failure in activation, and decreased torque development speed are widely acknowledged in the literature^54,55^. Quadriceps and hamstring muscles are frequently co-contracted by ACLR patients to stabilize the knee^56^. These differences showed that the clinicians should pay more attention on the athletes with ACLR to prevent further injuries. They may prescribe the rehabilitation programs in longer durations and more functional manner to decrease these differences. But we should keep in mind that the current study is a cross sectional study, also we are so not sure if ACLR patients had this strategy before their ACLR or not.

Individuals who have undergone ACLR displayed increased co-activation during functional activities. More specifically, voluntary activation of the quadriceps muscles during knee extension revealed heightened co-contraction of the medial and lateral hamstring muscles, suggesting that this co-contraction might hinder the mechanical efficiency of the quadriceps in those with ACLR^57^. The co-activation of the hamstrings in relation to the quadriceps is crucial for knee stability, aiming to decrease the tensile force applied to the ACL or graft tissue^56^. Insights from experiments involving simulated work on cadaveric knees^58^ have demonstrated that higher co-activation of the hamstring muscles correlates with reduced strain on the ACL. Consequently, these findings suggest that elevated levels of co-activation could represent an adaptive strategy to better counteract anterior tibial shear and rotation during functional tasks, promoting dynamic knee stability. Recent research supports this, showing that individuals with increased co-activation were less susceptible to graft rupture^59^. Likewise, other researchers^60^ found analogous connections between intralimb muscle strength (specifically the hamstring-to-quadriceps ratio) and the risk of graft rupture, collectively suggesting the relevance of intralimb muscle function and balance in preventing secondary injuries.

Interestingly, during everyday activities like walking and climbing stairs, co-activation levels were notably 2.75% to 10.72% higher. However, this increase was not observed in more dynamic athletic tasks such as double-limb or single-limb landings. This suggests that individuals with ACLR might struggle to effectively transfer this compensatory pattern to sports activities where enhanced knee stability is desirable. On the contrary, elevated co-activation can lead to greater compressive forces around the knee and limited flexion–extension movement during dynamic tasks^61,62^. This represents an unfavorable outcome of this strategy during repetitive daily activities, since these limitations have been associated with cartilage degeneration and the development of post-traumatic knee osteoarthritis^61,63^. Additionally, heightened co-activation of the medial hamstring has been linked to increased loading on the medial tibiofemoral joint, potentially contributing to the observed higher incidence of post-traumatic osteoarthritis in the medial compartment^64^.

We should consider that the prevalence of recurrent ACL rupture is high, and ranges from 1 to 11%^2^. Additionally, the prevalence of secondary contralateral ACL injury is as high as 1 in 4^3^, which suggests an elevated risk of ACL injury among athletes who have undergone a, ACLR^4–6^. Even with a significant volume of research underscoring the significance of consistent evaluation of quadriceps strength following ACLR, nearly half of clinicians persist in relying solely on the duration since surgery as the primary factor to determine an individual's readiness to resume sports activities after ACLR^65^. While the period of 6–9 months post-surgery is frequently suggested for returning to competitive sports, the majority of athletes might not yet have achieved the physical readiness needed to perform at an elevated level^65^.

## Limitations

This study has some limitations. For instance, the group of individuals who had undergone ACLR were assessed at the 12-month mark post-surgery. It remains unclear whether these irregular movement patterns normalize over time, which could provide insights into determining the optimal duration required for a successful return to sports (RTS). Furthermore, a considerable portion of the ACLR group in this study received a medial hamstring autograft. The research was conducted exclusively on dominant leg of female athletes, and its findings might differ for non-dominant leg or inactive women. Similarly, the outcomes of the study may exhibit variations within the community of male athletes. Although these findings are novel, they are only generalizable to single-leg drop landing and cannot be extrapolated to double-leg drop landing. Finally, this study had no objective data on patient reported outcomes and limb symmetry metrics that may be effective factors to explain the findings.

## Conclusion

This research has presented initial indications that, one year following ACLR, a tendency for the quadriceps to avoid engagement in the reconstructed limb results in diminished knee joint power generation and a tendency to protect the knee during a SLL task. Recognizing that strength is a trainable physical attribute, medical professionals should prioritize the restoration of quadriceps strength in post-ACLR patients and assess their strength regularly throughout the rehabilitation process. Additionally, heightened co-activation of the medial hamstring has been linked to increased loading on the medial tibiofemoral joint, potentially contributing to the observed higher incidence of post-traumatic osteoarthritis in the medial compartment.

### Supplementary Information

Supplementary Information.

## Data Availability

The raw data and material will be available online after publishing the paper as a supplementary file 1 in the journal. More queries will reply by corresponding author in future.

## References

[1] Beynnon BD, Johnson RJ, Abate JA, Fleming BC, Nichols CE (2005). Treatment of anterior cruciate ligament injuries, part I. Am. J. Sports Med..

[2] Gans I, Retzky JS, Jones LC, Tanaka MJ (2018). Epidemiology of recurrent anterior cruciate ligament injuries in National Collegiate Athletic Association sports: The Injury Surveillance Program, 2004–2014. Orthop. J. Sports Med..

[3] Paterno MV, Rauh MJ, Thomas S, Hewett TE, Schmitt LC (2022). Return-to-sport criteria after anterior cruciate ligament reconstruction fail to identify the risk of second anterior cruciate ligament injury. J. Athl. Train..

[4] Samitier G, Marcano AI, Alentorn-Geli E, Cugat R, Farmer KW, Moser MW (2015). Failure of anterior cruciate ligament reconstruction. Arch. Bone Jt. Surg..

[5] Nawasreh ZH, Yabroudi MA, Anan A-S, Daradkeh S, Kassas M, Bashaireh K (2022). Kinetic energy absorption differences during drop jump between athletes with and without radiological signs of knee osteoarthritis: Two years post anterior cruciate ligament reconstruction. Gait Posture.

[6] Georgoulis JD, Melissaridou D, Patras K, Megaloikonomos PD, Trikoupis I, Savvidou OD (2023). Neuromuscular activity of the lower-extremities during running, landing and changing-of-direction movements in individuals with anterior cruciate ligament reconstruction: A review of electromyographic studies. J. Exp. Orthop..

[7] Abourezk MN, Ithurburn MP, McNally MP, Thoma LM, Briggs MS, Hewett TE (2017). Hamstring strength asymmetry at 3 years after anterior cruciate ligament reconstruction alters knee mechanics during gait and jogging. Am. J. Sports Med..

[8] Markström JL, Liebermann DG, Schelin L, Häger CK (2022). Atypical lower limb mechanics during weight acceptance of stair descent at different time frames after anterior cruciate ligament reconstruction. Am. J. Sports Med..

[9] Somerville V, Bringans C, Braakhuis A (2017). Polyphenols and performance: A systematic review and meta-analysis. Sports Med..

[10] Maestroni, L., Turner, A., Papadopoulos, K., Pedley, J., Sideris, V. & Read, P. Single leg drop jump is affected by physical capacities in male soccer players following ACL reconstruction. *Sci. Med. Football* (2023) **(just-accepted)**. 10.1080/24733938.2023.2225481 37314868

[11] Smeets A, Verschueren S, Staes F, Vandenneucker H, Claes S, Vanrenterghem J (2021). Athletes with an ACL reconstruction show a different neuromuscular response to environmental challenges compared to uninjured athletes. Gait Posture.

[12] Trigsted SM, Post EG, Bell DR (2017). Landing mechanics during single hop for distance in females following anterior cruciate ligament reconstruction compared to healthy controls. Knee Surg. Sports Traumatol. Arthrosc..

[13] Ithurburn MP, Paterno MV, Ford KR, Hewett TE, Schmitt LC (2015). Young athletes with quadriceps femoris strength asymmetry at return to sport after anterior cruciate ligament reconstruction demonstrate asymmetric single-leg drop-landing mechanics. Am. J. Sports Med..

[14] Norcross MF, Lewek MD, Padua DA, Shultz SJ, Weinhold PS, Blackburn JT (2013). Lower extremity energy absorption and biomechanics during landing, part I: Sagittal-plane energy absorption analyses. J. Athl. Train..

[15] Sahrmann S (2017). The how and why of the movement system as the identity of physical therapy. Int. J. Sports Phys. Ther..

[16] Sheikhhoseini R, Kavianifard M, Hoseini Nejad SE, Piri H (2020). Comparison of the mechanical energy transfer of gait in female athletes with and without non-specific chronic low back pain. Women’s Health Bull..

[17] Gholami F, Letafatkar A, Moghadas Tabrizi Y, Gokeler A, Rossettini G, Ghanati HA (2023). Comparing the effects of differential and visuo-motor training on functional performance, biomechanical, and psychological factors in athletes after ACL reconstruction: A randomized controlled trial. J. Clin. Med..

[18] Vittersø AD, Halicka M, Buckingham G, Proulx MJ, Bultitude JH (2022). The sensorimotor theory of pathological pain revisited. Neurosci. Biobehav. Rev..

[19] Kristjansson E, Treleaven J (2009). Sensorimotor function and dizziness in neck pain: Implications for assessment and management. J. Orthop. Sports Phys. Ther..

[20] Page, P., Frank, C. C. & Lardner, R. Assessment and treatment of muscle imbalance (2010).

[21] Shimizu T, Samaan MA, Tanaka MS, Pedoia V, Souza RB, Li X (2019). Abnormal biomechanics at 6 months are associated with cartilage degeneration at 3 years after anterior cruciate ligament reconstruction. Arthroscopy.

[22] Marin MI, Popescu D, Burileanu AH, Rusu L (2023). Dynamic Functional Stability Analysis of Gait After Anterior Cruciate Ligament (ACL) Reconstruction. International Workshop on Medical and Service Robots.

[23] Ithurburn MP, Paterno MV, Thomas S, Pennell ML, Evans KD, Magnussen RA (2019). Change in drop-landing mechanics over 2 years in young athletes after anterior cruciate ligament reconstruction. Am. J. Sports Med..

[24] Ali N, Robertson DGE, Rouhi G (2014). Sagittal plane body kinematics and kinetics during single-leg landing from increasing vertical heights and horizontal distances: Implications for risk of non-contact ACL injury. Knee.

[25] McGibbon CA, Krebs DE, Puniello MS (2001). Mechanical energy analysis identifies compensatory strategies in disabled elders’ gait. J. Biomech..

[26] Lee J-H, Cynn H-S, Kwon O-Y, Yi C-H, Yoon T-L, Choi W-J (2014). Different hip rotations influence hip abductor muscles activity during isometric side-lying hip abduction in subjects with gluteus medius weakness. J. Electromyogr. Kinesiol..

[27] Khan W, Nokes L, Jones R, Johnson D (2007). The relationship of the angle of immobilisation of the knee to the force applied to the extensor mechanism when partially weight-bearing: A gait-analysis study in normal volunteers. J. Bone Jt. Surg. Br..

[28] Di Stasi SL, Hartigan EH, Snyder-Mackler L (2012). Unilateral stance strategies of athletes with ACL deficiency. J. Appl. Biomech..

[29] Hewett TE, Myer GD, Ford KR, Heidt RS, Colosimo AJ, McLean SG (2005). Biomechanical measures of neuromuscular control and valgus loading of the knee predict anterior cruciate ligament injury risk in female athletes: A prospective study. Am. J. Sports Med..

[30] Ward SH, Blackburn JT, Padua DA, Stanley LE, Harkey MS, Luc-Harkey BA (2018). Quadriceps neuromuscular function and jump-landing sagittal-plane knee biomechanics after anterior cruciate ligament reconstruction. J. Athl. Train..

[31] Aleshinsky SY (1986). An energy ‘sources’ and ‘fractions’ approach to the mechanical energy expenditure problem—I. Basic concepts, description of the model, analysis of a one-link system movement. J. Biomech..

[32] Aleshinsky SY (1986). An energy ‘sources’ and ‘fractions’ approach to the mechanical energy expenditure problem—II. Movement of the multi-link chain model. J. Biomech..

[33] Aleshinsky SY (1986). An energy ‘sources’ and ‘fractions’ approach to the mechanical energy expenditure problem—V. The mechanical energy expenditure reduction during motion of the multi-link system. J. Biomech..

[34] Aleshinsky SY (1986). An energy ‘sources’ and ‘fractions’ approach to the mechanical energy expenditure problem—III. Mechanical energy expenditure reduction during one link motion. J. Biomech..

[35] Kotsifaki A, Van Rossom S, Whiteley R, Korakakis V, Bahr R, Sideris V (2022). Single leg vertical jump performance identifies knee function deficits at return to sport after ACL reconstruction in male athletes. Br. J. Sports Med..

[36] Ernst GP, Saliba E, Diduch DR, Hurwitz SR, Ball DW (2000). Lower-extremity compensations following anterior cruciate ligament reconstruction. Phys. Ther..

[37] Graham MC, Reeves KA, Johnson DL, Noehren B (2023). Relationship between quadriceps strength and knee joint power during jumping after ACLR. Orthop. J. Sports Med..

[38] Goerger BM, Marshall SW, Beutler AI, Blackburn JT, Wilckens JH, Padua DA (2015). Anterior cruciate ligament injury alters preinjury lower extremity biomechanics in the injured and uninjured leg: The JUMP-ACL study. Br. J. Sports Med..

[39] Davies GJ, McCarty E, Provencher M, Manske RC (2017). ACL return to sport guidelines and criteria. Curr. Rev. Musculoskelet. Med..

[40] Roe C, Jacobs C, Hoch J, Johnson DL, Noehren B (2022). Test batteries after primary anterior cruciate ligament reconstruction: A systematic review. Sports Health.

[41] Romanchuk NJ, Del Bel MJ, Benoit DL (2020). Sex-specific landing biomechanics and energy absorption during unanticipated single-leg drop-jumps in adolescents: Implications for knee injury mechanics. J. Biomech..

[42] Nagi SZ (1991). Disability concepts revisited; implications for prevention. Disability in America Toward a National Agenda for Prevention.

[43] Pope AM, Brandt EN (1997). Enabling America: Assessing the Role of Rehabilitation Science and Engineering.

[44] Jette AM, Assmann SF, Rooks D, Harris BA, Crawford S (1998). Interrelationships among disablement concepts. J. Gerontol. Ser. A Biol. Sci. Med. Sci..

[45] Hannon JP, Wang-Price S, Goto S, Singleton S, Dietrich L, Bothwell J (2021). Twelve-week quadriceps strength as a predictor of quadriceps strength at time of return to sport testing following bone-patellar tendon-bone autograft anterior cruciate ligament reconstruction. Int. J. Sports Phys. Ther..

[46] Turk R, Shah S, Chilton M, Thomas TL, Anene C, Mousad A (2023). Critical Criteria Recommendations: Return to Sport After ACL reconstruction requires evaluation of time after surgery of 8 months, > 2 functional tests, psychological readiness, and quadriceps/hamstring strength. Arthroscopy.

[47] Singleton S, Scofield H, Davis B, Waller A, Garrison C, Goto S (2023). Altered knee loading following primary ACL repair versus ACL reconstruction. Int. J. Sports Phys. Ther..

[48] Judd DL, Dennis DA, Thomas AC, Wolfe P, Dayton MR, Stevens-Lapsley JE (2014). Muscle strength and functional recovery during the first year after THA. Clin. Orthop. Relat. Res..

[49] Rivera-Brown AM, Frontera WR, Fontánez R, Micheo WF (2022). Evidence for isokinetic and functional testing in return to sport decisions following ACL surgery. PM&R..

[50] Ingersoll CD, Grindstaff TL, Pietrosimone BG, Hart JM (2008). Neuromuscular consequences of anterior cruciate ligament injury. Clin. Sports Med..

[51] Hart JM, Pietrosimone B, Hertel J, Ingersoll CD (2010). Quadriceps activation following knee injuries: A systematic review. J. Athl. Train..

[52] Nyland J, Caborn D, Rothbauer J, Kocabey Y, Couch J (2003). Two-year outcomes following ACL reconstruction with allograft tibialis anterior tendons: A retrospective study. Knee Surg. Sports Traumatol. Arthrosc..

[53] Riemann BL, Lephart SM (2002). The sensorimotor system, part I: The physiologic basis of functional joint stability. J. Athl. Train..

[54] Scheurer SA, Sherman DA, Glaviano NR, Ingersoll CD, Norte GE (2020). Corticomotor function is associated with quadriceps rate of torque development in individuals with ACL surgery. Exp. Brain Res..

[55] Norte GE, Hertel J, Saliba SA, Diduch DR, Hart JM (2018). Quadriceps neuromuscular function in patients with anterior cruciate ligament reconstruction with or without knee osteoarthritis: A cross-sectional study. J. Athl. Train..

[56] Hurd WJ, Snyder-Mackler L (2007). Knee instability after acute ACL rupture affects movement patterns during the mid-stance phase of gait. J. Orthop. Res..

[57] Sherman DA, Glaviano NR, Norte GE (2021). Hamstrings neuromuscular function after anterior cruciate ligament reconstruction: A systematic review and meta-analysis. Sports Med..

[58] Withrow TJ, Huston LJ, Wojtys EM, Ashton-Miller JA (2008). Effect of varying hamstring tension on anterior cruciate ligament strain during in vitro impulsive knee flexion and compression loading. J. Bone Jt. Surg. Am..

[59] Palmieri-Smith RM, Strickland M, Lepley LK (2019). Hamstring muscle activity after primary anterior cruciate ligament reconstruction—A protective mechanism in those who do not sustain a secondary injury? A preliminary study. Sports Health.

[60] Kyritsis P, Bahr R, Landreau P, Miladi R, Witvrouw E (2016). Likelihood of ACL graft rupture: Not meeting six clinical discharge criteria before return to sport is associated with a four times greater risk of rupture. Br. J. Sports Med..

[61] Blackburn T, Pietrosimone B, Goodwin JS, Johnston C, Spang JT (2019). Co-activation during gait following anterior cruciate ligament reconstruction. Clin. Biomech..

[62] MacWilliams B, Wilson D, DesJardins J, Romero J, Chao E (1999). Hamstrings cocontraction reduces internal rotation, anterior translation, and anterior cruciate ligament load in weight-bearing flexion. J. Orthop. Res..

[63] Khandha A, Manal K, Wellsandt E, Capin J, Snyder-Mackler L, Buchanan TS (2017). Gait mechanics in those with/without medial compartment knee osteoarthritis 5 years after anterior cruciate ligament reconstruction. J. Orthop. Res..

[64] Flaxman TE, Alkjær T, Simonsen EB, Krogsgaard MR, Benoit DL (2017). Predicting the functional roles of knee joint muscles from internal joint moments. Med. Sci. Sports Exerc..

[65] Burgi CR, Peters S, Ardern CL, Magill JR, Gomez CD, Sylvain J (2019). Which criteria are used to clear patients to return to sport after primary ACL reconstruction? A scoping review. Br. J. Sports Med..

